# Reply to Saher, L. Outcomes of Microvascular Decompression Surgery: A Study on Cerebrospinal Fluid Leakage and Surgical Techniques. Comment on “Lee, H.S.; Park, K. Dura Closure Tactics to Prevent CSF Leakage in Microvascular Decompression Surgery. *Life* 2025, *15*, 574”

**DOI:** 10.3390/life15071056

**Published:** 2025-07-01

**Authors:** Hyun Seok Lee, Kwan Park

**Affiliations:** Department of Neurosurgery, Konkuk University Medical Center, Seoul 05030, Republic of Korea

Thank you for your valuable comments and thoughtful suggestions [1]. We also acknowledge that the three-layer sealing (sandwich) method and fleece-bound tissue sealing technique you mentioned are excellent methods for dural closure [2]. Ideally, achieving a watertight dura closure without the use of any material would be the most desirable way. However, in posterior fossa surgeries, this is often technically challenging. Although our previous “plugging muscle technique” already demonstrated a sufficiently low rate of cerebrospinal fluid (CSF) leakage, we aimed to further reduce the risk of subdural infection by avoiding the placement of any material inside the subdural space [3]. To address the risk of subdural infection, we developed and adopted a new closure technique that provides a good prognosis of CSF leakage without introducing any foreign material into the subdural space.

We follow a previously published diagnostic algorithm for suspected CSF leaks, which utilizes radiologic examinations, including temporal bone CT, clinicopathologic examination, and the patient’s clinical presentation. Applying this protocol to a cohort of 475 patients, we observed no cases requiring reoperation due to CSF leakage, which we considered to be a good diagnostic practice [4]. Therefore all patients undergo routine postoperative follow-up in the outpatient clinic, allowing for the continuous monitoring of long-term outcomes related to cerebrospinal fluid (CSF) leakage. To date, no cases of delayed CSF leakage have been observed.

We also appreciate your thoughtful observation concerning the economic considerations associated with the technique. Compared to the sandwich technique, our method—using TachoSil over a 7.5 × 7.5 cm piece of DuraGen—may be considered relatively more cost-effective in the context of the Korean healthcare system. In Korea, some materials are covered by the National Health Insurance system, and our method is not more costly compared to the sandwich technique that applies TachoSil to both the inner and outer dura. Nevertheless, we acknowledge that cost considerations may vary depending on the healthcare system and reimbursement policies in other countries. We appreciate the important point about the lack of a direct control group. Nevertheless, given the wealth of existing research on dural closure methods in posterior fossa surgery, we consider that an indirect comparison with the prognostic outcomes of our technique could reasonably be made.

We sincerely appreciate your thoughtful and detailed appraisal of our article, “Dura Closure Tactics to Prevent CSF Leakage in Microvascular Decompression Surgery.” Your comments provide valuable insights and raise important points that we fully acknowledge and agree merit further exploration.
